# Oomycetes found in wild and cultivated areas of Vietnam

**DOI:** 10.3389/fmicb.2026.1606112

**Published:** 2026-02-04

**Authors:** Emily E. Pfeufer, Glen Groben, Lindsay Harrison, Pham Quang Thu, Timothy Widmer, Alina S. Puig

**Affiliations:** 1 United States Department of Agriculture, Agricultural Research Service, Foreign Disease Weed Science Research Unit, Frederick, MD, United States; 2 Oak Ridge Institute for Science and Education, Agricultural Research Service Research Participation Program, Oak Ridge, TN, United States; 3 Vietnamese Academy of Forest Sciences, Hanoi, Vietnam

**Keywords:** baiting, forests, genetic barcoding, natural ecosystems, rivers, soilborne oomycetes, Southeast Asia

## Abstract

To determine the diversity of oomycetes in Vietnam, particularly in the presumed center of origin of most *Phytophthora* taxa, isolates were collected from rivers, agricultural soils, and forested areas. Species identification was performed using sequences from the internal transcribed spacer (ITS) and cytochrome 2 oxidase (*cox2*) regions of the genome. Of the 245 isolates included in this study, the majority (66.5%) were identified as *Phytopythium* spp., followed by *Phytophthora* spp. (31%) and *Pythium* spp. (2.4%). The most prevalent species were *Phytopythium vexans* and *Phytophthora cinnamomi*, accounting for 51.8 and 24.5%, respectively, of all isolates obtained. A total of 17 isolates were identified as belonging to multiple undescribed species. From agricultural soils, only one isolate each of *Phytophthora* and *Pythium* was obtained, with the remaining 93% belonging to the genus *Phytopythium*. This study shows that natural and agricultural areas in Vietnam harbor a wide diversity of oomycetes, including several undescribed species. The identification of oomycete species in a center of origin will help identify potential emerging pathogens that can become a threat to U.S. agriculture.

## 1 Introduction

The oomycete group includes over 1,500 species, encompassing organisms that infect aquatic lifeforms, such as *Saprolegnia,* as well as pathogens of forest and food crops (Judelson and Ah-Fong, 2019; Thines, 2018; Kamoun et al., 2015). Oomycetes that affect plants include the genera *Phytophthora*, *Phytopythium*, and *Pythium,* as well as numerous unculturable genera that cause downy mildew disease. The number of recognized oomycete species has dramatically increased in recent years. Approximately 75% of *Phytophthora* species have been formally described since 2004, and the number of *Phytopythium* species has doubled since the genus was established in 2010 (Jung et al., 2024; Bala et al., 2010).

*Phytophthora*, *Phytopythium*, and *Pythium* are morphologically similar, possessing hyaline, coenocytic mycelia (Ho, 2018; Ghimire and Baysal-Gurel, 2023). *Phytopythium* species were classified within *Pythium* clade K until 2010, when they were reassigned to an independent genus (Bala et al., 2010). *Phytopythium* and *Pythium* produce zoospores in a similar manner, with undifferentiated protoplasm released from the sporangia through a tube into a vesicle, followed by differentiation into zoospores (Marano et al., 2014; Ho, 2018; Nam and Choi, 2019). In *Phytophthora*, the differentiation into zoospores occurs entirely within the sporangium (Ho, 2018).

Multiple surveys of oomycetes have been conducted in Southeast Asia, as evidence suggests that it is the center of origin for *Phytophthora* clades 2, 5, 6, 7, 8, and 9 (Dang et al., 2021; Jung et al., 2017; Jung et al., 2020; Zentmeyer, 1988). Large-scale collection efforts have also been undertaken on additional continents to improve the understanding of diversity and evolution within the genus (Jung et al., 2024). Despite rapid taxonomic advancements, our understanding of *Phytophthora* may only encompass 55% of the total species (Scott et al., 2019).

Numerous surveys have been conducted in natural areas of Southeast Asia; however, none have included agricultural areas. In Vietnam, *Phytophthora* species have not been found causing disease in forest environments (Jung et al., 2017), but several species are known to cause fruit rot and gummosis in citrus plants, as well as root rot in black pepper within the country (Van Tran et al., 2023; Puglisi et al., 2017; Thao et al., 2024). The objective of this study was to determine the oomycetes present in natural and agricultural areas of Vietnam by sampling river water and soil from both forests and agricultural regions (Table 1). The knowledge gained from this research opens avenues for a more comprehensive understanding of undescribed oomycetes and potential connections between uncultivated and cultivated areas.

**Table 1:** Summary of sites in Vietnam sampled for oomycetes in 2018.

Substrate	Prevalent plant(s)	Elevation (above sea level)	Unique sites	Isolates
River water	Natural forest	43–2,275 m	24	35
Soil	Natural forest	3–2,259 m	55	180
Soil	Citrus	10–47 m	9	20
Soil	Longan	15–18 m	2	6
Soil	*Cinnamomum cassia*	356–558 m	3	4

## 2 Materials and methods

### 2.1 Sampling and isolation

Isolates used in this study were collected from multiple locations across Vietnam in 2018, as summarized in Table 1 and displayed geographically in Supplementary Figure 1. Elevation, substrate, prevalent plants, province, and geographic coordinates were recorded and maintained as part of the dataset.

Two methods were used to isolate oomycetes based on the type of the sample taken. Soil samples (10 cc) were collected in disposable cups and brought to the laboratory, where they were flooded with sterile water and kept at 18–20 °C under natural light. Pieces of leaflets aged 3–10 days from *Lithocarpus bacgiangensis*, *Quercus glauca*, *Q. gilva*, *Castanopsis indica*, *Chamaecyparis hodginsii*, or *Acacia mangium* were placed on the flooded soil, where they served as baits for newly emerging zoospores. After 2–3 days, a subset of the leaflets developed necrotic lesions characteristic of *Phytophthora* infection. Lesion margins were surface-disinfected, air-dried, and plated onto PARPNH agar, as described by Erwin and Ribeiro (1996). PARPNH consists of 20% V8 agar containing 10 mg pimaricin, 200 mg ampicillin, 10 mg rifampicin, 200 mg pentachloronitrobenzene, 50 mg nystatin, and 50 mg hymexazol per liter (Jung et al., 2000). PARPNH is semi-selective for oomycetes. For river samples, water was collected, and leaves of *Castanopsis* sp. or *Quercus* sp. were placed on the surface of the water to serve as baits. Following the development of necrotic lesions, the organisms were isolated and processed as described above.

Resulting colonies were subcultured by transferring small fragments of mycelia to obtain organisms in pure culture. Isolates were stored at 4 °C on the colonized plates of PARPNH agar and annually subcultured to maintain viability.

### 2.2 DNA extraction and PCR

To extract genomic DNA, isolates were grown in pure culture on clarified 20% V8 juice agar for 1 week. Aerial mycelium and the upper layer of the media plate were gently scraped with a sterile scalpel, and genomic DNA was extracted using the FastDNA soil extraction kit (FastDNA, MP Biomedicals, Solon, OH). The genomic DNA was used as a template in standard PCR reactions using internal transcribed spacer (ITS) 6/4 (White et al., 1990; Cooke et al., 2000) and *cox2* primers (Hudspeth et al., 2000). PCR products were visualized by gel electrophoresis to confirm amplification based on the presence of 1 kb and 640 bp bands for ITS and *cox2*, respectively.

### 2.3 Isolate identification

PCR amplicons were purified using ExoSap-IT (Applied Biosystems; Thermo Fisher Scientific, Carlsbad, CA, USA) and submitted for Sanger sequencing (Eurofins Inc., Louisville, KY, USA). Initial reactions were sequenced with the ITS6 primer, but the samples that yielded low-quality sequences were additionally sequenced in the reverse direction using the ITS4 primer. In these cases, forward and reverse sequences were aligned, edited, and analyzed using BLASTn. To confirm or clarify identification, amplified fragments of the *cox*2 region were bidirectionally sequenced, aligned, edited, and analyzed with BLASTn.

### 2.4 Phylogenetic analyses

**Figure 1:**
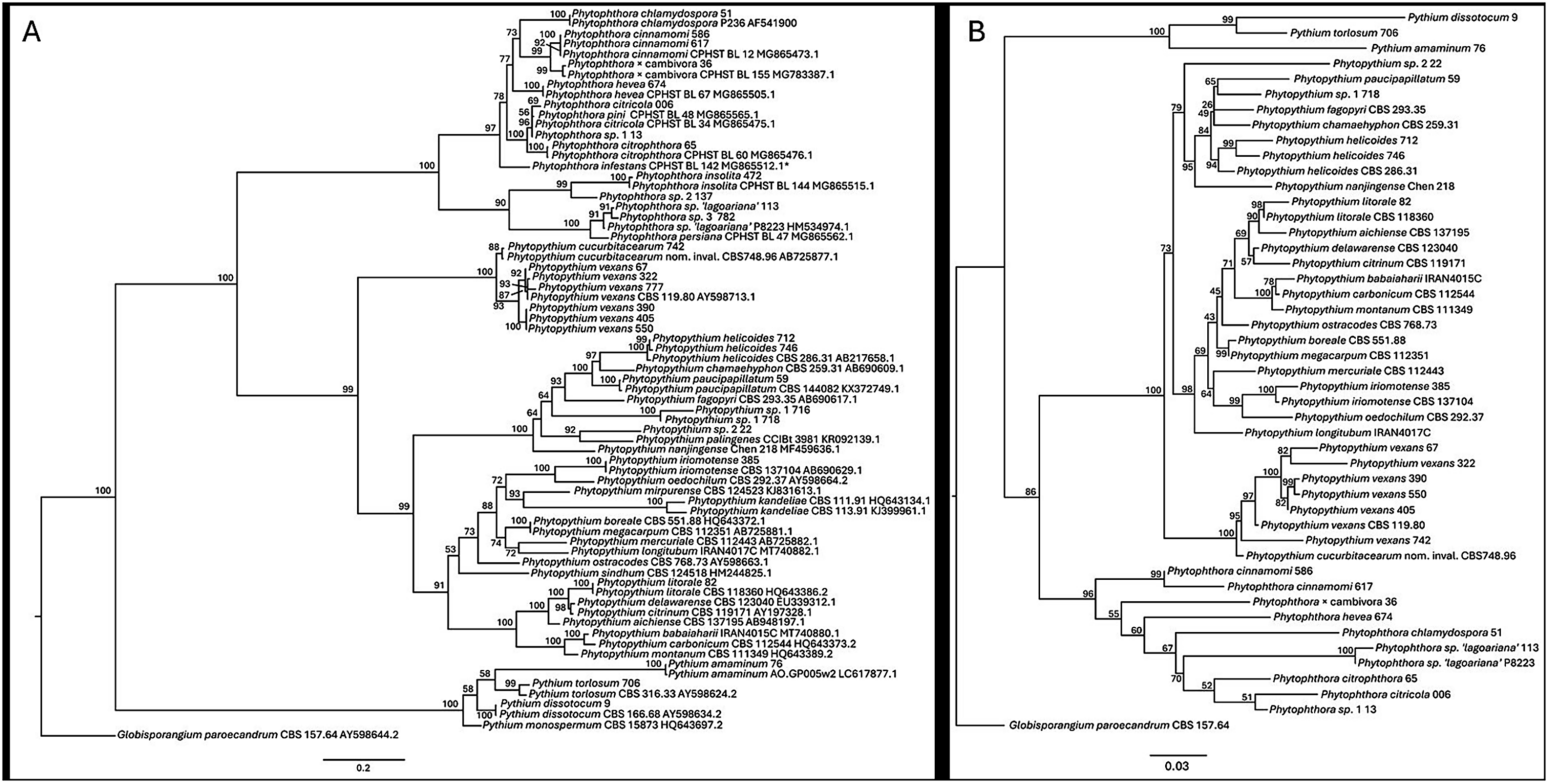
Phylogenetic trees of the ITS **(A)** and *cox*2 **(B)** sequences of the *Phytopythium*, *Phytophthora,* and *Pythium* isolates obtained in this study. Isolates with labels ending in a 1- to 3-digit number are from the current study, while other isolates used in the comparison in the phylogenetic tree are publicly available sequences of reference strains.

To determine the diversity of oomycetes detected in this study, phylogenetic analyses were conducted using sequences of the rDNA internal transcribed spacer (ITS) and the cytochrome c oxidase 2 subunit (*cox*2). The ends of each sanger sequence were trimmed using the Trim End function in Geneious Prime (2023.0.1; Biomatters Inc., Boston, MA, USA) with the corresponding primer pair. When available, a consensus sequence was generated from each bidirectional sequence for both ITS and *cox*2 using the *De Novo* Assemble function in Geneious Prime. Representative sequences of known species were retrieved from GenBank for *Phytopythium*, *Phytophthora*, *Pythium,* and the outgroup *Globisporangium paroecandrum* (Supplementary Table 1). The representative sequences were aligned with the Vietnam isolate sequences using MAFFT (v7.490) (Katoh and Standley, 2013), and terminal regions, including primer regions, were trimmed using Geneious Prime. Sequences with missing ends were replaced with “?” to denote missing data. The resulting alignment was analyzed with IQ-TREE (v2.2.3) (Nguyen et al., 2015) to generate maximum likelihood trees with automatic model selection (Kalyaanamoorthy et al., 2017). The resulting Newick tree files were visualized using FigTree v1.4.4.^1^ The tree was rooted using the consensus sequence of *Globisporangium paroecandrum* CBS 157.64 (Figure 1). This process was done to generate separate ITS and *cox*2 gene trees. A concatenated tree was not generated because most reference *Phytophthora* and *Pythium* sequences do not have *cox*2 sequences.

Sequences were deposited in NCBI GenBank: ITS accessions OR531129–OR531278 and *cox2* accessions OR704574–OR704733. Isolates showing >99% sequence identity to a deposited reference sequence were classified as that species, provided that these groups were supported by phylogenetic analysis.

## 3 Results

### 3.1 Oomycetes found in agricultural and forest areas of Vietnam

**Figure 2:**
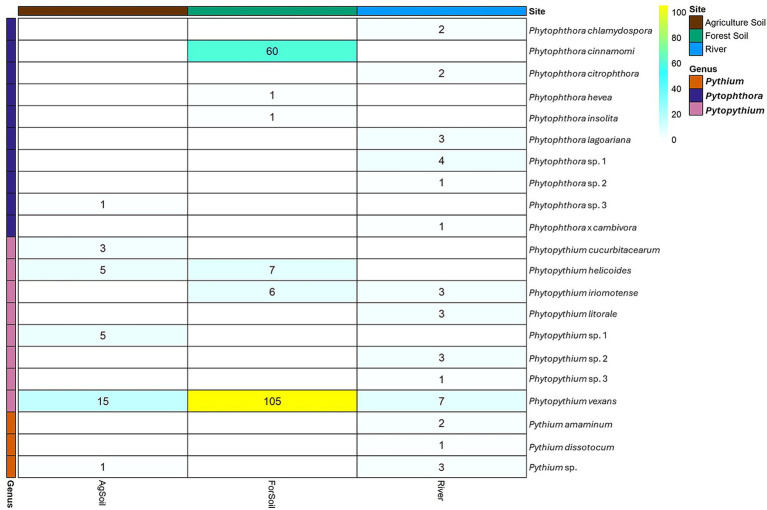
Heatmap summarizing oomycete taxa obtained from the different types of sites (agricultural soil, forest soil, and water).

Over 700 isolates were obtained from the 93 sites sampled in 2018; however, less than half were revived following long-term storage. Sequences were generated from 245 isolates, corresponding to 21 unique oomycete species. The majority of isolates (66.5%) were identified as *Phytopythium* spp., followed by *Phytophthora* spp. (31%) (Table 2) and *Pythium* spp. (2.4%). *Phytopythium* spp. accounted for 93.3% (28 of 30) of isolates obtained from agricultural soils, 65.6% (118 of 180) from forest soils, and 48.6% (17 of 35) from river samples. Oomycete taxa obtained from the different types of sites (agricultural soil, forest soil, and water) are summarized in a heatmap (Figure 2).

**Table 2:** Subgeneric taxonomic identifications of oomycete isolates from Vietnam, based on >99% ITS DNA sequence homology and phylogenetic grouping.

Genus	Species	Number of isolates (*n* = 245)	Origin^b^
*Phytopythium* (*n* = 163)	*vexans*	127	Soil_F, River, Soil_L, Soil_Ct, Soil_Cn
	*helicoides*	12	Soil_F, Soil_Ct
	*iriomotense*	9	Soil_F, River
	*littorale*	3	River
	*cucurbitacearum*	3	Soil_Ct
	*paucipapillatum*	1	River
	sp. 1	5	Soil_Ct
	sp. 2	3	River
*Pythium* (*n* = 6)	*dissotocum*	1	River
	*amaminum*	2	River
	sp. (nr *torlosum*)	3	River, Soil_Ct
*Phytophthora* (*n* = 76)	*cinnamomi*	60	Soil_F
	*chlamydospore*	2	River
	*citrophthora*	2	River
	*hevea*	1	Soil_F
	*insolita*	1	Soil_F
	*lagoariana*	3	River
	*x cambivora* y	1	River
	sp. 1 (nr^a^ pini)	4	River
	sp. 2 (nr plurivora)	1	River
	sp. 3 (nr lagoariana)	1	Soil_Cn

a Nr = Near. This abbreviation indicates the species to which the isolate is most closely related based on the results from BLASTn analysis.

b Substrate origin of isolates. River: Symptomatic leaves collected from natural rivers in forested areas; Soil_F: Soil samples collected from natural forests; Soil_L: Soil samples collected from sites predominantly planted with longan; Soil_Ct: Soil samples collected from sites predominantly planted with citrus; and Soil_Cn: Soil samples collected from sites predominantly planted with *Cinnamomum cassia*.

*Phytopythium vexans* and *Phytophthora cinnamomi* were the most frequently isolated species, accounting for 77.9 and 78.9% of isolates within their respective genera (Figure 2). Just over 6.9% (17 out of 245) of sequenced isolates could not be assigned to a species due to either having high (>98%) nucleotide similarity to multiple GenBank entries purportedly representing different species or not sharing high similarity with any entries. Among *Phytopythium* taxa, *Pp. vexans*, which was recovered from all three site types, was the most frequently isolated species (77.9%), followed by *Pp. helicoides* (7.6%) and *Pp. iriomotense* (5.7%). Species-level identification could not be made for nine *Phytopythium* isolates originating from citrus soil and river water, and they are labelled here as *Phytopythium* sp. 1 (*n* = 5) and *Phytopythium* sp. 2 (*n* = 3) (Table 2).

*Phytopythium* sp. 2 shared 99% identity with sequences of undescribed *Phytopythium* species previously reported by Jung et al. (2020) in rivers in Vietnam (MN872739) and with another isolate obtained from forest soil in Poland (KC602492). BLASTn analysis of the *cox*2 sequences showed the closest match to *Pp. helicoides* (MN952211); however, they only shared 96.7% nucleotide identity, which is below the threshold expected for a member of the same species, based on other isolates in this study. A total of 11 *Phytophthora* taxa were obtained, with five represented by a single isolate.

Among the isolated *Phytophthora* spp., most isolates (*n* = 60; 78.9%) belonged to *P. cinnamomi*, followed by *P. lagoariana* (*n* = 3) and *P. pini* (*n* = 3). The majority of *Phytophthora* isolates were obtained from forest soils (81.6%), with only 17.1 and 1.3% from the river and agricultural soil samples, respectively. A total of six isolates appeared to belong to three undescribed species of *Phytophthora*, with the ITS sequence of *P.* sp. 1 grouping equally closely with *P. pini* and *P. citricola* (Figure 1). *P.* sp. 2 shared the highest nucleotide identity (92.3%) with a sequence of *P. plurivora* (KT693124) from forest soil in Hungary, followed by 92.2% shared identity with a sequence of *P. polonica* (KT693123). *P.* sp. 3 showed greater than 99% sequence identity with numerous taxa, including two putative hybrids: *P*. sp. zentmyerii × Peru4-like from Portugal (PQ238050) and *P*. sp. × Kunnunara-like from Taiwan (KU682602).

*Pythium amaminum* (*n* = 2) and *Py. dissotocum* (*n* = 1) were isolated from river water, while a third species, *Py.* sp. (*n* = 3), was isolated from both river water and citrus soil. This unidentified species could not be confirmed based on GenBank matches and may represent an undescribed species. In the phylogenetic analysis, it clustered closely with the reference isolate *Py. torulosum* (CBS 166.68_AY598634), despite 23 mismatches and 14 gaps between the two sequences (Figure 1). The three isolates of the unknown *Pythium* species shared ≥99.6% identity in *cox*2 sequences and ≥99.3% identity in *ITS* sequences. These high levels of sequence similarity justified their classification within the same species.

### 3.2 Phylogenetic analysis

The ITS alignment included 72 sequences (32 from this study) with 1,309 columns, 1,007 distinct patterns, 666 parsimony-informative sites, 147 singleton sites, and 495 constant sites. The best-fit model was TIM3 + F + I + G4. The ITS sequences of *Phytopythium*, *Phytophthora*, and *Pythium* spp. obtained in this study grouped with well-established reference isolates (Figure 1A). The two new *Phytopythium* species grouped with members of clades 2, such as *Pp. fagopyri* and *Pp. palingenes*. Among the undescribed *Phytophthora* species, *P.* sp. 1 clustered within clade 2, while *P.* sp. 2 and 3 fell within clade 9. The single undescribed *Pythium* species isolated grouped with *Py. torulosum,* which is part of clade B of this genus.

The *cox*2 alignment contained 47 sequences with 594 columns, 248 distinct patterns, 180 parsimony-informative sites, 58 singleton sites, and 356 constant sites. The best-fit model was TPM2u + F + I + R3. Few reference isolates had *cox*2 sequences available, so these were not included in the second phylogenetic analysis (Figure 1B). Despite this, the *cox*2 and ITS trees shared similar architecture (Figure 1).

## 4 Discussion

Vietnam has been a focus of oomycete surveys, especially for members of the genus *Phytophthora* (Jung et al., 2020; Dang et al., 2021), which is why it was chosen for this study. Although the original goal was to identify potentially new *Phytophthora* species, most isolates obtained in this study belonged to the *Phytopythium* genus. The majority of these were the known pathogen *Pp. vexans* (Baten et al., 2014), which was also reported in a similar oomycete-focused survey in Taiwan (Jung et al., 2017).

The high prevalence of *Phytopythium* spp. in this study is consistent with the reports of oomycete surveys conducted in agricultural areas (Parke et al., 2019; Navarro-Acevedo et al., 2021; Beluzan et al., 2022). Following collection, the isolates obtained in this study were maintained in culture for three years before being analyzed; therefore, *Phytopythium* may also have been better at surviving long-term storage.

The composition of *Phytophthora*, *Phytopythium*, and *Pythium* communities obtained via baiting may be influenced by the production practices of the agricultural fields sampled, as well as conditions during the baiting process, such as incubation temperature (Navarro-Acevedo et al., 2021). In addition, the use of a baiting step selects for plant-associated oomycete species, which may only comprise a fraction of all species present (Redekar et al., 2019). Unculturable species, such as *P. cyperi* (syn. *Kawakamia cyperi* [Thines et al., 2023]) and *P. lepironiae*, which can only survive within plant hosts, would not be detected in these studies (Jung et al., 2024; Bourret et al., 2025). In addition, some *Phytophthora* spp. produce few or no zoospores and may therefore be underrepresented when using soil baiting, the sole method used in the current study for soil isolations.

*Phytopythium helicoides*, one of the most well-known plant pathogens in the genus (Rezaei et al., 2021), was found in both agricultural and forest soils. New reports of *Pp. helicoides* pathogenicity have rapidly increased since the early 2010s across a wide range of hosts (Yang et al., 2014; Wang et al., 2015; Marin et al., 2019), including impactful stem rot on citrus (Chen et al., 2016). Commercial citrus production soils made up the majority of agricultural soil samples in the present study. However, verification of the pathogenicity of these isolates on plant hosts, particularly citrus, is needed to further demonstrate the potential impact of *Pp. helicoides* and other members of Pp clade 2 on agricultural systems.

*Phytophthora* spp. comprised the majority of non-*Phytopythium* isolates in the collection, especially members of clade 7 (Abad et al., 2023). The majority (61 of 66) of clade 7 isolates shared close (98% or more) *cox*2 identity with *P. cinnamomi*. This species has one of the broadest host ranges among all plant pathogens, a cosmopolitan distribution, and Southeast Asia has been suggested as its center of diversity (Shakya et al., 2021). One clade 7 *Phytophthora* hybrid, *P.* x *cambivora*, was recovered from river water; aquatic environments have been suggested by others as a *Phytophthora* hybridization court (Burgess, 2015; Oh et al., 2013; Yang et al., 2014).

A greater number of taxa were recovered from the river samples, with over twice as many taxa as were found in forest and agricultural samples. Results from Kageyama et al. (2022) suggest that phytopathogenic oomycetes naturally inhabit rivers, which can then serve as important pathogen transmission routes to agricultural areas, where they cause disease outbreaks. For example, *Pp. litorale*, the pathogen responsible for root rot of apple (*Malus domestica*) and plane (*Platanus orientalis*), has previously been found at frequencies of up to 100% in irrigation water (Derviş et al., 2020; Mert et al., 2020; Redekar et al., 2019; Parkunan and Ji, 2013).

The high prevalence of *Phytopythium* in agricultural soils in this study could indicate an adaptation toward pathogenicity on crop plants. However, it could also be due to adaptation to the environmental and soil conditions created by agricultural practices. Land use has been found to affect numerous parameters, including the physicochemical properties of soil. Some studies have reported that natural forests have higher pH than cultivated lands (Muche et al., 2015), while the opposite pattern has been found in other studies (Dupouey et al., 2002; Ritter et al., 2003). Boie et al. (2024) found that oomycete species were significantly correlated with soil pH and organic content. They found higher species diversity in medium-textured soils compared to light or heavy soils. Although pH was not measured at the sites included in the study, the connection between pH and oomycete community composition is an area that warrants additional investigation.

This study shows that natural and agricultural areas in Vietnam harbor a wide diversity of oomycetes, including several undescribed species. Numerous undescribed species were detected, including a species of *Phytopythium* identified at multiple citrus production sites. The prevalence of *Phytopythium* spp. also suggests that its impact on tropical agricultural production areas may be underestimated. Further studies aimed at characterizing novel species and examining their roles as saprophytes versus pathogens will provide valuable information on potential emerging pathogens.

## Data Availability

The datasets presented in this study can be found in online repositories. The names of the repository/repositories and accession number(s) can be found in the article/Supplementary material.

## Footnotes

1
http://tree.bio.ed.ac.uk/software/figtree/
