# Supplementary material for: Oomycetes found in wild and cultivated areas of Vietnam
Source: Front Microbiol. 2026 Feb 4;17:1606112. doi: 10.3389/fmicb.2026.1606112 (PMC12913451; doi:10.3389/fmicb.2026.1606112)
Supplement: Supplementary file 1 [file Table_1.DOCX]

Supplementary Material

# Supplementary Figures and Tables

## Supplementary Figures


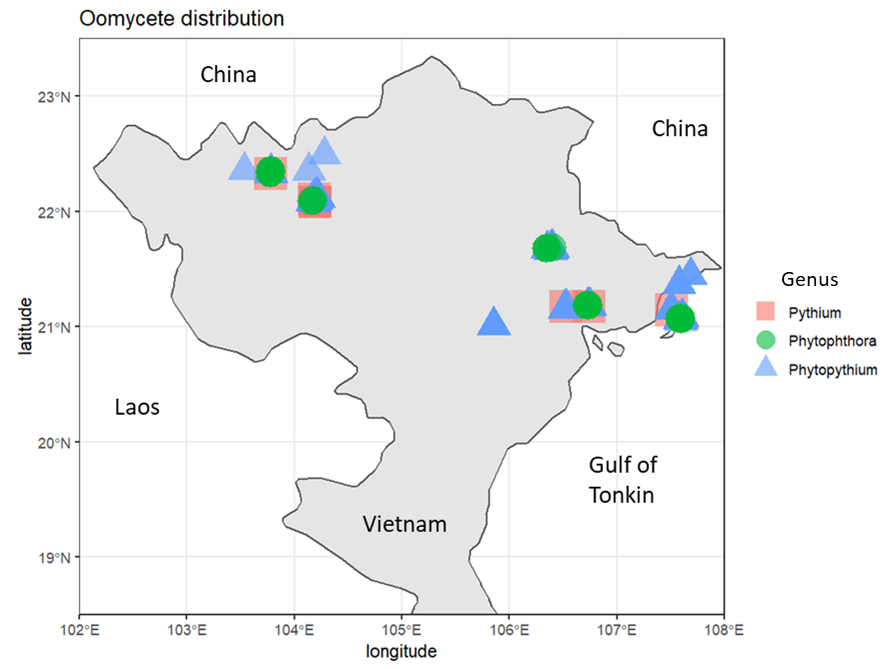


**Supplementary Figure 1.** Geographic location of sample sites in Vietnam from which *Pythium*, *Phytophthora*, and *Phytopythium* species were obtained in 2018.

## Supplementary Tables

**Supplementary Table 1.** Reference sequences used in phylogenetic analyses.

| Species | Isolate | Type | ITS | cox2 |
| --- | --- | --- | --- | --- |
| *Phytopythium aichiense* | CBS137195 | Type | AB948197 | AB948192 |
| *Phytopythium babaiaharii* | IRAN4015C | Type | MT740880.1 | MT720670.1 |
| *Phytopythium boreale* | CBS551.88 |  | AB725879.1 | AB690677.1 |
| *Phytopythium carbonicum* | CBS112544 |  | AB725876 | AB690678 |
| *Phytopythium chamaehyphon* | CBS259.30 | Type | AB690609 | AB690674 |
| *Phytopythium citrinum* | CBS119171 |  | AY197328 | AB690679 |
| *Phytopythium delawarense* | CBS 123040 | Type | EU339312.1 | KJ595430.1 |
| *Phytopythium dogmae* | USTCMS 4101 | Type |  | MF359561.1 |
| *Phytopythium fagopyri* | CBS293.35 |  | AB690617 | AB690671 |
| *Phytopythium helicoides* | CBS286.31 |  | AB725878 | AB690675 |
| *Phytopythium indigoferae* | CBS 261.30 |  | AY598714.1 |  |
| *Phytopythium iriomotense* | CBS137104 |  | AB690629 | AB690689 |
| *Phytopythium kandeliae* | CBS113.91 |  | KJ399961.1 |  |
| *Phytopythium kandeliae* | CBS111.91 |  | HQ643134.1 |  |
| *Phytopythium leanoi* | USTH 4102 | Type |  | MF359560.1 |
| *Phytopythium litorale* | CBS118360 | Type | DQ144637.1 | KJ595418.1 |
| *Phytopythium longitubum* | IRAN 4017C | Type | MT740882.1 | MT720672 |
| *Phytopythium megacarpum* | CBS112351 |  | AB725881 | AB690665 |
| *Phytopythium mercuriale* | CBS122443 | Type | AB725882 | AB690666 |
| *Phytopythium mirpurense* | CBS124523 | Type | KJ831613 |  |
| *Phytopythium montanum* | CBS111349 | Type | AB725883 | AB690667 |
| *Phytopythium nanjingense* | Chen 218 |  | MF459636 | MG788317 |
| *Phytopythium oedochilum* | CBS292.37 |  | AB690619 | AB690676 |
| *Phytopythium ostracodes* | CBS768.73 |  | AY598663 | AB690668 |
| *Phytopythium palingenes* | CCIBt 3981 |  | KR092139 |  |
| *Phytopythium paucipapillatum* | CBS 144082 | Type | KX372749 |  |
| *Phytopythium sindhum* | CBS124518 | Type | HM244825.1 |  |
| *Phytopythium vexans* | CBS 119.80 |  | AY598713.1 | EF426547.1 |
| *Phytopythium cucurbitacearum nom. inval* | CBS748.96 |  | AB725877.1 | AB690680.1 |
| *Pythium amaminum* | AO.GP005w2 | Type | LC617877.1 |  |
| *Pythium torlosum* | CBS 316.33 |  | AY598624.2 |  |
| *Pythium dissotocum* | CBS 166.68 |  | AY598634.2 |  |
| *Pythium monospermum* | CBS 15873 |  | HQ643697.2 |  |
| *Phytophthora chlamydospora* | P236 |  | AF541900.1 |  |
| *Phytophthora cinnamomi* | CPHST BL 12 | Type | MG865473.1 |  |
| *Phytophthora × cambivora* | CPHST BL 155 |  | MG783387.1 |  |
| *Phytophthora hevea* | CPHST BL 67 | Type | MG865505.1 |  |
| *Phytophthora pini* | CPHST BL 48 | Type | MG865565.1 |  |
| *Phytophthora citricola* | CPHST BL 34 | Type | MG865475.1 |  |
| *Phytophthora citrophthora* | CPHST BL 60 | Type | MG865476.1 |  |
| *Phytophthora infestans* | CPHST BL 142 |  | MG865512.1 |  |
| *Phytophthora insolita* | CPHST BL 144 | Type | MG865515.1 |  |
| *Phytophthora sp. 'lagoariana’* | P8223 |  | HM534974.1 | HM534974.1 |
